# Development of a pseudo-typed virus particle based method to determine the efficacy of virucidal agents

**DOI:** 10.1038/s41598-024-52177-2

**Published:** 2024-01-25

**Authors:** Jordan Thomas, Farah Mughal, Kelly J. Roper, Aurelia Kotsiri, Wejdan Albalawi, Abdullateef Alshehri, Yugandhar B. S. Reddy, Sayandip Mukherjee, Georgios Pollakis, William A. Paxton, Michael Hoptroff

**Affiliations:** 1 Department of Clinical Infection, Microbiology and Immunology (CIMI), Institute of Infection, Veterinary and Ecological Sciences (IVES), University of Liverpool, Liverpool, L69 7BE UK; 2 Unilever Research & Development Centre, 64 Main Road, Whitefield, Bangalore, Karnataka 560066 India; 3 Unilever Research & Development, Port Sunlight, Bebington, Wirral CH63 3JW UK

**Keywords:** Microbiology, Virology, SARS-CoV-2, Microbiology techniques

## Abstract

The ongoing Severe Acute Respiratory Syndrome Coronavirus 2 (SARS-CoV-2) pandemic has highlighted the threat that viral outbreaks pose to global health. A key tool in the arsenal to prevent and control viral disease outbreaks is disinfection of equipment and surfaces with formulations that contain virucidal agents (VA). However, assessment of the efficacy of virus inactivation often requires live virus assays or surrogate viruses such as Modified Vaccinia Virus Ankara (MVA), which can be expensive, time consuming and technically challenging. Therefore, we have developed a pseudo-typed virus (PV) based approach to assess the inactivation of enveloped viruses with a fast and quantitative output that can be adapted to emerging viruses. Additionally, we have developed a method to completely remove the cytotoxicity of virucidal agents while retaining the required sensitivity to measure PV infectivity. Our results indicated that the removal of cytotoxicity was an essential step to accurately measure virus inactivation. Further, we demonstrated that there was no difference in susceptibility to virus inactivation between PVs that express the envelopes of HIV-1, SARS-CoV-2, and Influenza A/Indonesia. Therefore, we have developed an effective and safe alternative to live virus assays that enables the rapid assessment of virucidal activity for the development and optimization of virucidal reagents.

## Introduction

The increasing trend towards globalization, coupled with the effects of accelerating climate change, has resulted in an alarming increase in the rate of emergence of novel infectious diseases^1–3^. In particular, zoonotic viral pathogens have dominated recent human infectious disease outbreaks, including the 2002–2004 outbreak of Severe Acute Respiratory Syndrome Coronavirus (SARS-CoV)^4,5^, the 2016 and 2018 Ebola virus (EBOV) epidemics^6^, the 2015 outbreak of Zika virus (ZIKV) in Brazil^7^, the current SARS-CoV-2 pandemic^8,9^, and the more recent outbreak of monkeypox^10^. Viruses can persist for extended periods on contaminated surfaces, with estimates for SARS-CoV-2 ranging from 72 h at room temperature, 7 days on surgical masks, and up to a month on refrigerated and frozen products^11–14^. Additionally, SARS-CoV-2 RNA is readily detected in faeces and wastewater^15–17^, although the persistence of live virus in this medium remains to be determined^18^. Accordingly, infection prevention and reduction of germ transmission are key epidemiological factors in controlling community outbreaks. Everyday hygiene products such as hand and body cleansers (soaps and sanitizers) as well as surface cleaners (sprays and wipes) provide easily accessible and affordable interventions that can considerably reduce the abundance of germs in and around us^19–22^. Towards this end, there is a growing need to develop robust, high-throughput assays and methodologies to determine the germicidal efficacy of prototype formulations, thereby facilitating speed of innovation in consumer hygiene products.

Currently, European guidelines to measure the efficacy of virucidal agents recommend a stepwise approach, in which the initial phases of testing are designed to quantify virucidal activity against viruses in suspension, followed by assays to measure virucidal activity when viruses are dried onto various surfaces^23^. These assays often rely on live viruses^24–28^, however, this is expensive and time consuming and, for highly pathogenic organisms, requires access to high containment level facilities. To overcome this, Modified Vaccinia Virus Ankara (MVA) is commonly used as a surrogate of virus inactivation, as this is more resistant to virucidal agents than other enveloped viruses such as EBOV, ZIKV, SARS-CoV and Middle East Respiratory Syndrome Coronavirus (MERS-CoV)^29–31^. However, MVA infection is measured either by microscopic examination of cytopathic effects (CPE) 8 days following inoculation^29,30^, immunostaining for viral antigens^32^, or through the use of recombinant MVA-expressing markers such as GFP^33^. As such, the quantitative output of these assays can be cumbersome, expensive and time consuming.

Another potential alternative to live viruses for assays that measure virucidal activity are Pseudo-typed Virus Particles (PV). PVs are viruses that contain the core structure and protein load of a surrogate virus, typically Human Immunodeficiency Virus (HIV), Murine Leukaemia Virus (MLV) or Vesicular Stomatitis Virus (VSV), while expressing the envelope proteins of a different virus or virus variant^34,35^. Generally, PVs are produced by co-transfection of separate plasmids that express the viral backbone components, the envelope protein(s) of interest and, in some cases, a reporter gene construct. Therefore, PVs can infect tropism-matched target cells but lack the genetic information required for de novo replication, making them a safer alternative to replication competent virus. Further, the incorporation of a reporter gene construct into the PV, such as luciferase or fluorescent protein, enables straightforward and fast quantitation of PV infection. As such, PVs have been extensively used in the study of serological responses to emerging viruses^35–41^ as well as for the investigation of host cell interactions with viral envelope proteins^42^. Similarly, due to their safety and relative ease of use, PVs can be employed for high-throughput, quantitative analysis of virus inactivation^43–46^, enabling measurement of inhibitory concentrations and therefore providing a means to optimise the formulation of virucidal reagents. In many cases, however, products that provide virus inactivation are cytotoxic and require high starting dilutions to prevent cytotoxic effects that would affect quantitation.

In this study, we have developed an assay to measure the virucidal activity of four different commercially available everyday hygiene products against various enveloped PVs in suspension. We have also developed a method to remove the cytotoxicity of virucidal agents without over-diluting the PV at the expense of sensitivity, therefore enabling the quantitation of virus inactivation at high concentrations of virucidal compounds. As such, this is a relatively low-cost tool to determine the inhibitory concentrations of different virucidal products, allowing for the optimization of commercially available everyday hygiene product formulations.

## Methods

### Cell culture

All cell lines were maintained in Dulbecco’s Modified Eagles Medium (DMEM) supplemented with 10% heat-inactivated foetal bovine serum (FBS), 2 mM/ml L-glutamine and 100 U/ml penicillin with 100 mg/ml streptomycin (Pen/Strep), herein termed complete DMEM, in tissue culture conditions (37 °C and 5% CO_2_). HEK293T LentiX (obtained from Takara Bio) cells were used to produce PVs via transfection of the relevant expression plasmids and also to measure transduction with Influenza virus enveloped PV. HEK293T cells stably expressing human ACE2 and TMPRSS2, termed HEK293T ACE2 TMPRSS2 (obtained from the National Institute of Biological Standards and Control, NIBSC), were used to measure transduction of SARS-CoV-2 enveloped PV. Finally, TZMbl cells (obtained from NIBSC Centre for AIDS Reagents, CFAR), a commonly used cell line that expresses the HIV-1 receptors CD4 and CCR5 and contains a luciferase reporter under the control of HIV-1 Tat, were used to measure HIV-1 enveloped PV.

### Plasmids

All plasmids were produced through heat-shock transformation of Top10 competent *E. coli* cells, eluted in molecular grade water and quantified using nanodrop spectrophotometry. For SARS-CoV-2 enveloped PV, the SARS-CoV-2 Spike (S) glycoprotein isolated from Wuhan early in the pandemic (Accession MN908947) was cloned into the pCDNA3.1 expression plasmid (produced by GeneArt Gene Synthesis). A plasmid expressing the envelope glycoprotein from vesicular stomatitis virus (VSVg), which exhibits a wide cell tropism range, was used as a positive control. A backbone plasmid expressing HIV-1 *gag-pol*, termed p8.91^47^, and a reporter construct that expresses luciferase, termed pCSFLW^48^, were used to form the core of SARS-CoV-2 (S) and VSVg enveloped PVs. For the production of HIV-1 enveloped PVs, a plasmid expressing the HIV-1 backbone deficient in Env (pSG3ΔEnv) and a plasmid expressing the HIV-1 LAI envelope were acquired from the HIV-1 Reagent Program. A plasmid expressing the Influenza A hemagglutinin (HA) envelope based on the A/Indonesia/5/2005 (H5) was a kind gift from Prof. Nigel Temperton and the plasmid used to express neuraminidase (NA) from Influenza A/Aichi/2/1968 (N2) was purchased from Stratech (VG40199-G-N-SIB).

### Pseudo-typed virus production

Single cycle infectious SARS-CoV-2 (S) and VSVg PV were produced by transfection of HEK293T LentiX cells according to a previously described protocol^49,50^. Briefly, 5.0 × 10^5^ HEK293T LentiX cells were seeded onto a tissue culture treated 6-well plate in 2 ml complete DMEM and incubated for 24 h. For transfection, 750 ng of pCSFLW, 500 ng of p8.91 and 450 ng of the SARS-CoV-2 (S) envelope plasmid were added to 100 µl of OptiMEM. For negative control PV, transfections were produced as described above but without envelope expression plasmids (ΔEnv). In a separate tube, 17.5 µl of 1 mg/ml polyethyleneimine (PEI) transfection reagent was added to 100 µl of OptiMEM and mixed well by vortexing. The OptiMEM/PEI solution was then added to the solution containing OptiMEM and plasmids and incubated for 20 min at room temperature, after which the solution was added dropwise per well of the 6-well plate containing HEK293T LentiX cells. Transfected cells were incubated overnight (maximum 16 h) under cell culture conditions. Following this incubation, the culture medium containing the transfection solution was replaced with complete DMEM and the cells were incubated for a further 48 h to allow PV production and egress. Finally, PV was harvested by filtration through a 0.45 µM syringe filter to remove cells or cell debris, after which the filtered PV was aliquoted and stored at −80 °C until use. Production of Influenza enveloped PV was performed as described above, with some alterations in the plasmids and quantities. In this case, 750 ng of pCSFLW, 500 ng of p8.91, 500 ng of Influenza A/Indonesia HA expression plasmid and 450 ng of Influenza NA were used. Finally, for the production of HIV-1 PV, transfection was performed in 10 cm^2^ dishes using 3 × 10^6^ LentiX cells. Production was then similar to the above described but scaled up to a larger plate. Specifically, 70 µl of 1 mg/ml PEI was added to 400 µl of OptiMEM mixed. Next, 2000 ng of pSG3ΔEnv backbone plasmid and 1800 ng of HIV-1 LAI envelope plasmid were added to 400 µl OptiMEM and this was mixed with the diluted PEI.

### ELISA quantification of PV

Single cycle infectious PV was quantified using an ELISA targeting the HIV-1 capsid protein, p24, which is expressed in the backbone plasmids p8.91 and pSG3ΔEnv, and is therefore present in all PV including envelope negative controls (ΔEnv). Viral p24 was quantified using Aalto Bioreagents LTD p24 kit according to manufactures instructions.

### PV transduction

The infectivity of newly produced PV was measured by infection of tropism matched target cells; 293T was used for Influenza enveloped PV, 293T ACE2 TMPRSS2 was used for SARS-CoV-2 (S) and VSVg enveloped PV and TZMbl was used for HIV-1 enveloped PV. The day prior to infection, 1.5 × 10^4^ cells were seeded onto a tissue culture treated, opaque white 96-well microplate in 200 µl complete DMEM. The following day, all of the media was removed from wells that were to be infected and 100 µl of PV was added in triplicate. The plate was incubated for 6 h after which an additional 100 µl of complete DMEM was added to infected wells and the plate was incubated for 48 h under cell culture conditions. For negative controls, a condition where only complete DMEM was added (cell only control) as well as a condition where an envelope negative PV (ΔEnv) was added. After 48 h, luciferase activity was measured in a BMG Fluostar Fluorometer using the Promega Luciferase Assay System according to manufacturer’s instructions, with luminescence expressed as Relative Light Units (RLU).

### Virucidal agents

Four different virucidal agents were used in this study including UNI01, consisting of [C12-C16 Alkyl dimethyl benzyl ammonium chloride (BAC) 0.75%. We also used UNI02, consisting of [C12-C16 Alkyl dimethyl benzyl ammonium chloride (BAC) 1.5%]. Additionally, we used commercially available formulations including UNI03, a hand sanitizer containing 70% alcohol as the active ingredient and UNI04, a liquid handwash consisting of a mixed surfactant system of anionic sodium laureth sulfate and non-ionic cocamide monoethanolamine as virucidal actives.

### LDH assay

Cell cytotoxicity was measured by quantification of Lactate Dehydrogenase (LDH) production using the CyQUANT LDH Cytotoxicity Assay according to manufacturer’s instructions, testing serial dilutions of the substance that were made in PBS were made in PBS. Absorbance was measured at 490 nm and 680 nm, after which the absorbance at 680 nm was subtracted from the 480 nm absorbance for all measurements. The percentage cytotoxicity was determined using the following formula:

$$\frac{treated\, sample\, LDH-spontaneous\, LDH}{maximum\, LDH-spontaneous\, LDH}\times 100.$$

### Pseudo-typed virus particle based virucidal activity assay

To measure the virucidal activity of different compounds, serial 1/1 dilutions of the compounds were made in sterile PBS. Pseudo-typed virus to be tested was diluted in complete DMEM to achieve infectious doses that produce between 3.0 × 10^4^ and 2.0 × 10^5^ RLUs following infection. For conditions in which the cytotoxic compounds were removed by passing through a column: 114 µl PV was aliquoted into tubes and 16 µl of the compound dilutions or PBS (PV control) were added to the aliquots to achieve a product dilution of 1/8. The tubes were mixed by flicking, briefly centrifuged and incubated at rt for 30 min. During this time, Cytiva Microspin S-400 HR columns were prepared by centrifuging at 735×*g* for 1 min and flow through containing the resin storage buffer was discarded. After incubation, 100 µl of the PV deactivation solution was added to the column and centrifuged at 735×*g* for 2 min. The flow through containing PV was diluted 1/5 in complete DMEM and 200 µl of this was used to transduce target cells in duplicate. A condition in which cells were treated with complete DMEM was used as a negative control. For the condition where the mix was passed through the column twice to remove cytotoxicity, 100 µl of the flow through from the first column was added to the next column directly. After this, the PV was diluted and used to transduce cells as described above. For conditions in which cytotoxic compounds were not removed, the PV was first passed through a column to ensure that the results could be comparable between conditions. In this case, Cytiva Microspin S-400 HR were prepared as described above. Next, 100 µl of diluted PV was passed through the columns as previously described and the flow through was diluted in 350 µl complete DMEM. Following this, 50 µl of compound dilutions was added to the diluted PV flow through to achieve a final dilution of 1/10. As before, 200 µl was added to the cells in duplicate and incubated for 48 h, after which luciferase activity (RLU) was measured to determine the infectivity. In some cases, extra PV flow through was saved to measure PV concentration by ELISA. Virucidal activity was determined by calculating % inhibition based on the infectivity of the treated sample compared to the infectivity measured from the sample treated with the maximum virucidal agents (VA) dilution using the following formula:

$$\frac{treated\, PV\, RLU -cells\, only\, RLU}{treated\, maximum\, dilution\, RLU-cells\, only\, RLU}\times 100.$$

Virucidal activity was expressed as % infectivity which was calculated by subtracting the measured % inhibition from 100.

### Statistical analyses

Statistical analyses were performed in GraphPad Prism 8.0 software. P values < 0.05 are denoted with * and P values < 0.005 are denoted with **. In all cases, the statistical methods used are supplied in each figure legend. For the determination of cytotoxicity or virucidal activity inhibitory concentrations (IC_50_ or IC_90_), GraphPad Prism 8.0 software was used to plot % cell survival or infectivity against the concentration of VA and analysed using the simple linear regression (four parameter) function.

## Results

### Production of a panel of enveloped PV

Initially, we aimed to produce large stocks of different enveloped PVs that could be used for the duration of the study. Pseudo-typed virus particle stocks were produced in 293T LentiX cells and infectivity was measured by transduction of the corresponding cell type and subsequent measurement of luciferase activity in those cells compared to a PV produced at the same time that lacked envelope glycoprotein (ΔEnv). Additionally, luciferase activity was measured in cells that were not infected to determine the background of the assay. Further, a highly infectious envelope with a broad host cell tropism (VSVg) was used as a positive control for PV production. In all cases, in order to allow accurate determination of PV infectivity and to prevent saturation of RLU measurements, both undiluted PV and PV that was diluted 1/20 were used to infect cells. For the production of SARS-CoV-2 (S) enveloped PV, there was a significant difference in the luciferase activity between the undiluted SARS-CoV-2 (S) (P = 0.0016, Supplementary Fig. 1A), HIV-1 X4 (P = 0.0097, Supplementary Fig. 1B), Influenza A/Indonesia (P = 0.0093, Supplementary Fig. 1C) and ΔEnv PV, indicating production of a PV that specifically infected tropism matched target cells. For VSVg, there was significant difference between both the undiluted (P = 0.0287) and diluted PV (P = 0.0287) and the envelope negative control, indicating production of highly infectious PV (Supplementary Fig. 1D).

### Determination of cytotoxicity of virucidal reagents

**Figure 1:**
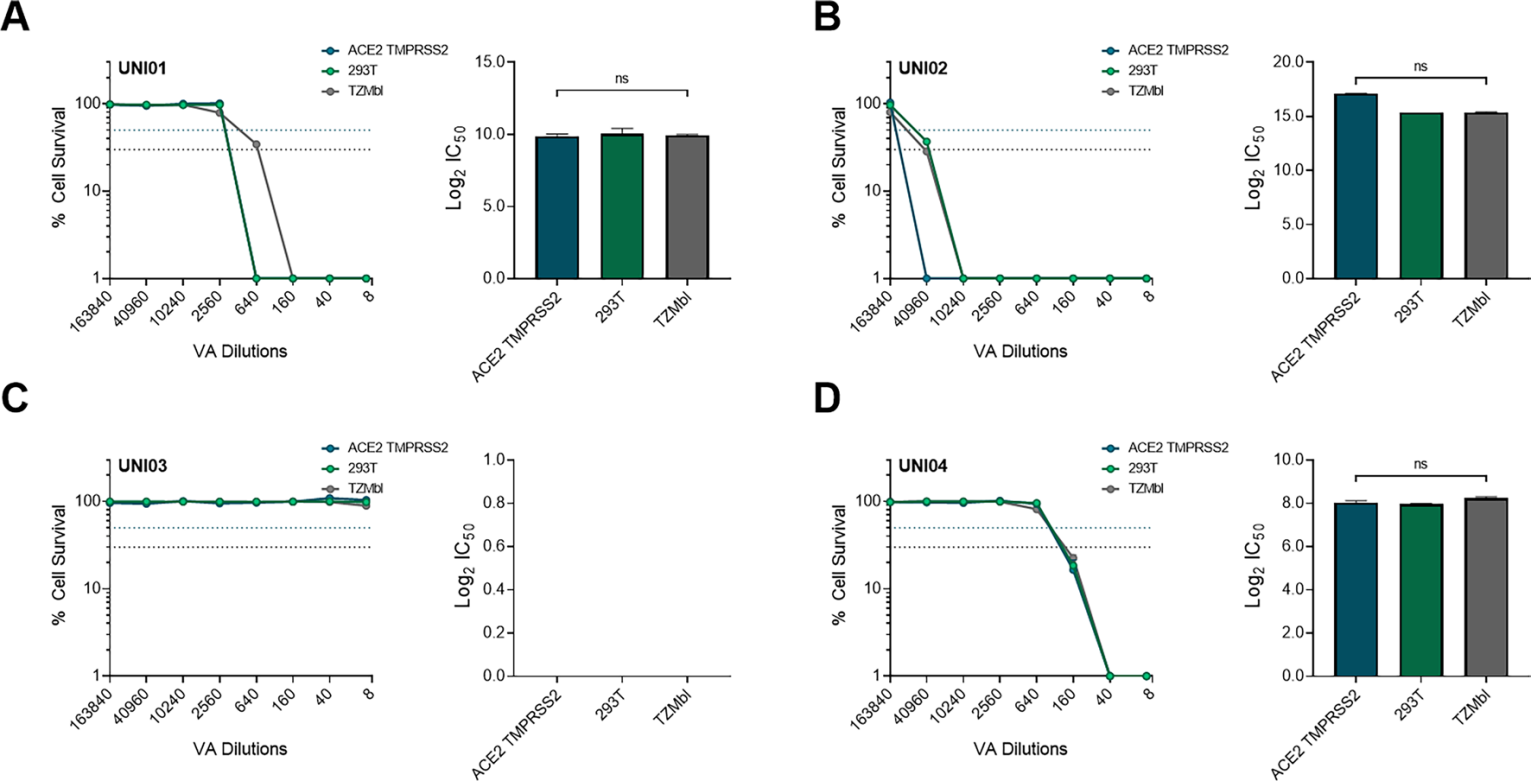
Measuring cytotoxicity of four virucidal reagents in three different cell types measured by LDH assay. In all cases, cytotoxicity was measured in 293T (green), ACE2 TMPRSS2 (blue) and TZMbl (grey). **(A)** Cytotoxicity curve of UNI01 and corresponding Log2 IC_50_ measurements for each cell type (n = 2). **(B)** Cytotoxicity curve of UNI02 and corresponding Log2 IC_50_ measurements for each cell type (n = 2). **(C)** Cytotoxicity curve of UNI03 and corresponding Log2 IC_50_ measurements for each cell type (n = 2). In this case, the reagent was not cytotoxic and so no corresponding Log2 IC_50_ could be determined and is therefore expressed as 0. **(D)** Cytotoxicity curve of UNI04 and corresponding Log2 IC_50_ measurements for each cell type (n = 2). Blue dotted line represents 50% cytotoxicity and black dotted line represents 70% cytotoxicity. Significant difference between IC_50_ values determined by Kruskal–Wallis test with Dunn’s multiple comparisons.

We next aimed to determine the cytotoxicity of each VA, including UNI01, UNI02, UNI03 and UNI04 in all three cell types that were used in this study. To this end, we serially diluted each product in PBS and added the product dilutions directly to the cells which were then incubated for 24 h. After this incubation, the LDH activity in each condition was measured and compared to a control in which lysis buffer was added to determine maximum LDH activity. For UNI01, the mean Log2 IC_50_ was 9.87 for ACE2 TMPRSS2 cells, 10.05 for 293T cells and 9.92 for TZMbl, indicating 50% cytotoxicity at a dilution of 1/938–1/1065 with no significant difference in the IC_50_ of any cell type (Fig. 1A). For UNI02, the Log2 IC_50_ was 17.11 for ACE2 TMPRSS2 cells, 15.36 for 293T cells and 15.39 for TZMbl, indicating 50% cytotoxicity at a dilution of 1/42,265–1/141,944 with no significant difference in the IC_50_ of any cell type (Fig. 1B). For UNI03, there was no observable cell death or LDH activity at any of the dilutions tested (Fig. 1C). For UNI04, the Log2 IC_50_ was 8.03 for ACE2 TMPRSS2 cells, 7.96 for 293T cells and 8.23 for TZMbl, indicating 50% cytotoxicity at a dilution of 1/249–1/301 with no significant difference in the IC_50_ of any cell type (Fig. 1D).

### Method for removal of cytotoxicity

**Figure 2:**
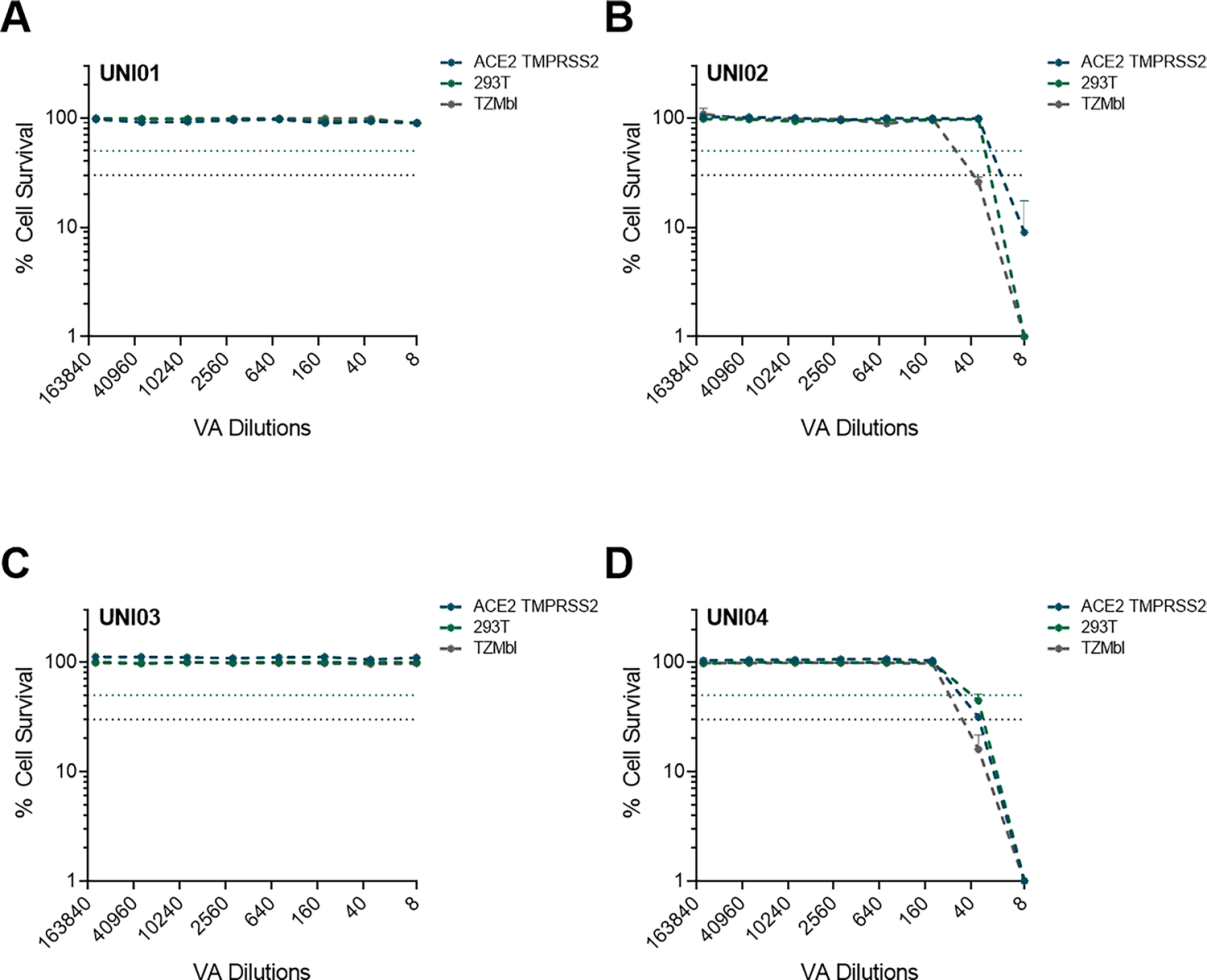
Cytotoxicity of antiviral reagents following treatment with Cytiva Microspin S-400 HR column. In all cases, cytotoxicity was measured in 293T (green), ACE2 TMPRSS2 (blue) and TZMbl (grey). **(A)** Cytotoxicity curve of UNI01 (n = 2). **(B)** Cytotoxicity curve of UNI02 (n = 2). **(C)** Cytotoxicity curve of UNI03 (n = 2). **(D)** Cytotoxicity curve of UNI04. Blue dotted line represents 50% cytotoxicity and black dotted line represents 70% cytotoxicity (n = 2).

The high cytotoxicity of UNI01, UNI02 and UNI04 was predicted to impede meaningful measurement of their virucidal activity and so we aimed to remove the cytotoxicity of the VAs prior to infection of target cells. To this end, we opted to use an approach in which 100 µl of VA was added to a Cytiva Microspin S-400 HR column, after which the 100 µl flow-through was diluted in 400 µl DMEM (1/5 final dilution) and subsequently added to the cells. Using this approach, we demonstrated complete removal of cytotoxicity for UNI01, resulting in 100% cell survival at the highest dilution tested (Fig. 2A). For UNI02, the majority of cytotoxicity was removed using this method, with 100% cell survival at ~ 1/40 dilution (Fig. 2B). Cytotoxicity was already negligible for UNI03 and so there was no change in the cell survival curve (Fig. 2C). Finally, we demonstrated nearly complete removal of cytotoxicity with UNI04, resulting in 100% cell survival at ~ 1/130 dilution (Fig. 2D).

Additionally, unlike replicative virus, PV is unable to undergo multiple rounds of infection or exponential replication that would be expected in live virus assays. As such, an important consideration for a PV based virucidal assay is the ability to retain viral titres that are high enough so that infection remains detectable. Therefore, we next aimed to determine the recovery of infectious PV following treatment with the microspin column, as measured by p24 ELISA and luciferase activity, respectively (Error! Reference source not found.). Through measurement of the total PV flow through via p24 ELISA, we demonstrated recovery of ~ 50% of input virus, with no significant difference between either untreated PV or PV that was recovered from the column (Suppl. Fig. 2A). Further, through measurement of luciferase activity of the PV flow through, we showed no significant difference in the infectiousness of the PV following Cytiva Microspin S-400 HR columns treatment when compared to untreated PV (Suppl. Fig. 2B). Based on these results, we proposed that this method was sensitive enough to measure the virucidal activity of different household products.

### Pseudo-typed virus particle based virucidal activity assay

**Figure 3:**
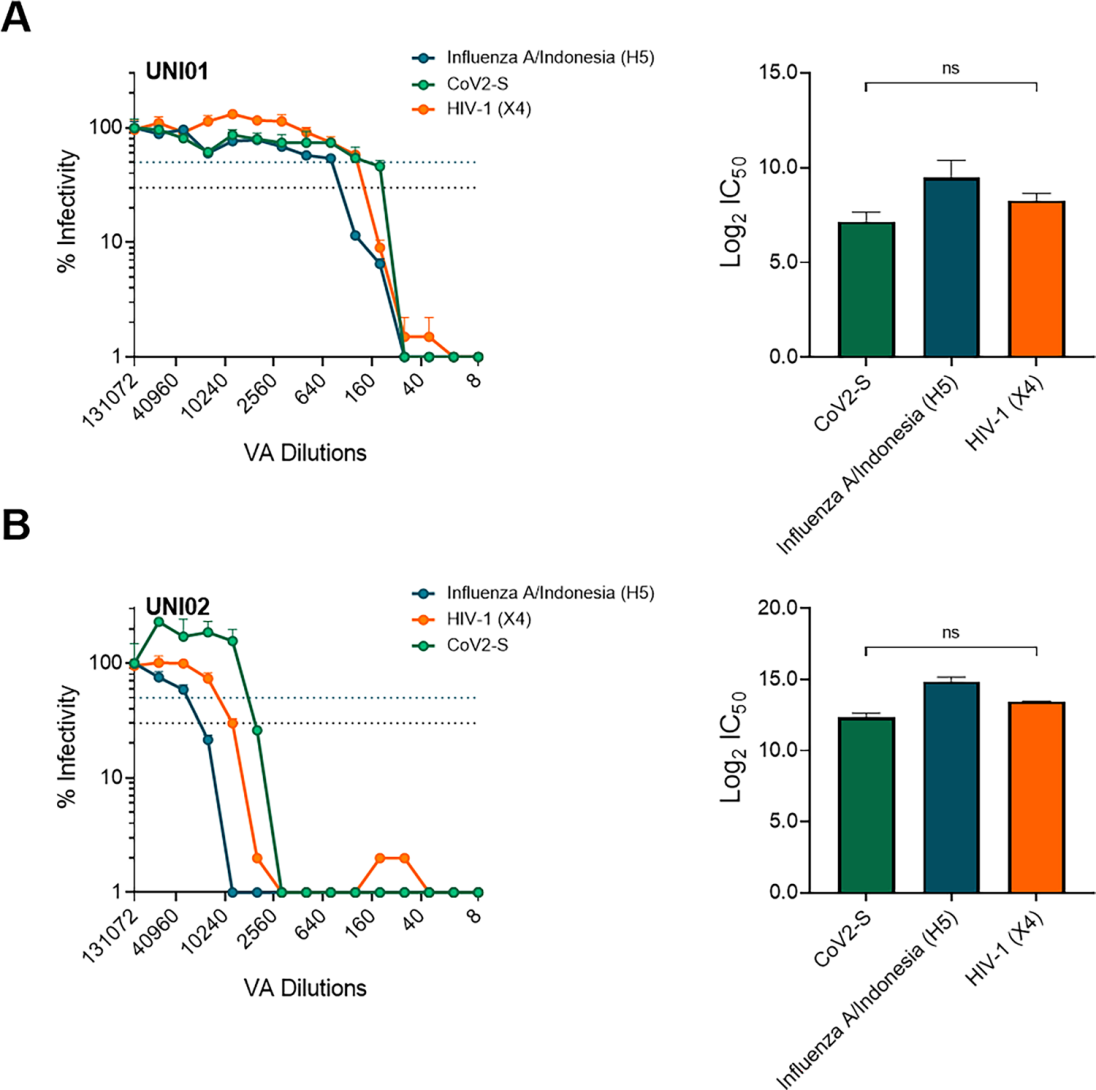
Measurement of virucidal activity of two different virucidal reagents. For these assays, Influenza A/Indonesia (H5) enveloped PV was transduced into 293T cells, SARS-CoV-2 enveloped PV was transduced into 293T ACE2 TMRPSS2 cells and HIV-1 (X4) was transduced into TZMbl cells. **(A)** Infectivity curve of three different enveloped PV and corresponding IC_50_ values when measuring the virucidal activity of UNI01 (n = 2). **(B)** Infectivity curve of three different enveloped PV and corresponding IC_50_ values when measuring the virucidal activity of UNI02. Blue dotted line represents 50% cytotoxicity and black dotted line represents 70% cytotoxicity (n = 2). Significant difference between IC_50_ values determined by Kruskal–Wallis test with Dunn’s multiple comparisons.

Following this, we aimed to determine the virucidal activity of two products, UNI01 and UNI02, on PVs that expressed three different envelopes including; SARS-CoV-2 (S), HIV-1 (X4) and Influenza A/Indonesia (H5). To this end, we serially diluted the VA in PBS up to 1/131,072 and incubated this with the PV for up to 30 min, after which the VA and PV mix was treated using a Cytiva Microspin S-400 HR as described in the previous section. Virucidal activity was determined by calculating the reduction in infectivity when compared with the infectivity of the maximum dilution. For UNI01, we observed a Log2 IC_50_ of 7.16 for CoV2-S, 9.49 for Influenza A/Indonesia (H5) and 8.23 for HIV-1 (X4), indicating 50% reduction in infectivity at 1/144–1/703 dilution, with no significant difference observed between different enveloped PVs (Fig. 3A). For UN02, we observed a Log2 IC_50_ of 12.33 for CoV2-S, 14.87 for Influenza A/Indonesia (H5) and 13.46 for HIV-1 (X4), indicating 50% reduction in infectivity at between 1/5164–1/29,865 dilution and with no significant differences observed between different enveloped PVs (Fig. 3B).

**Figure 4:**
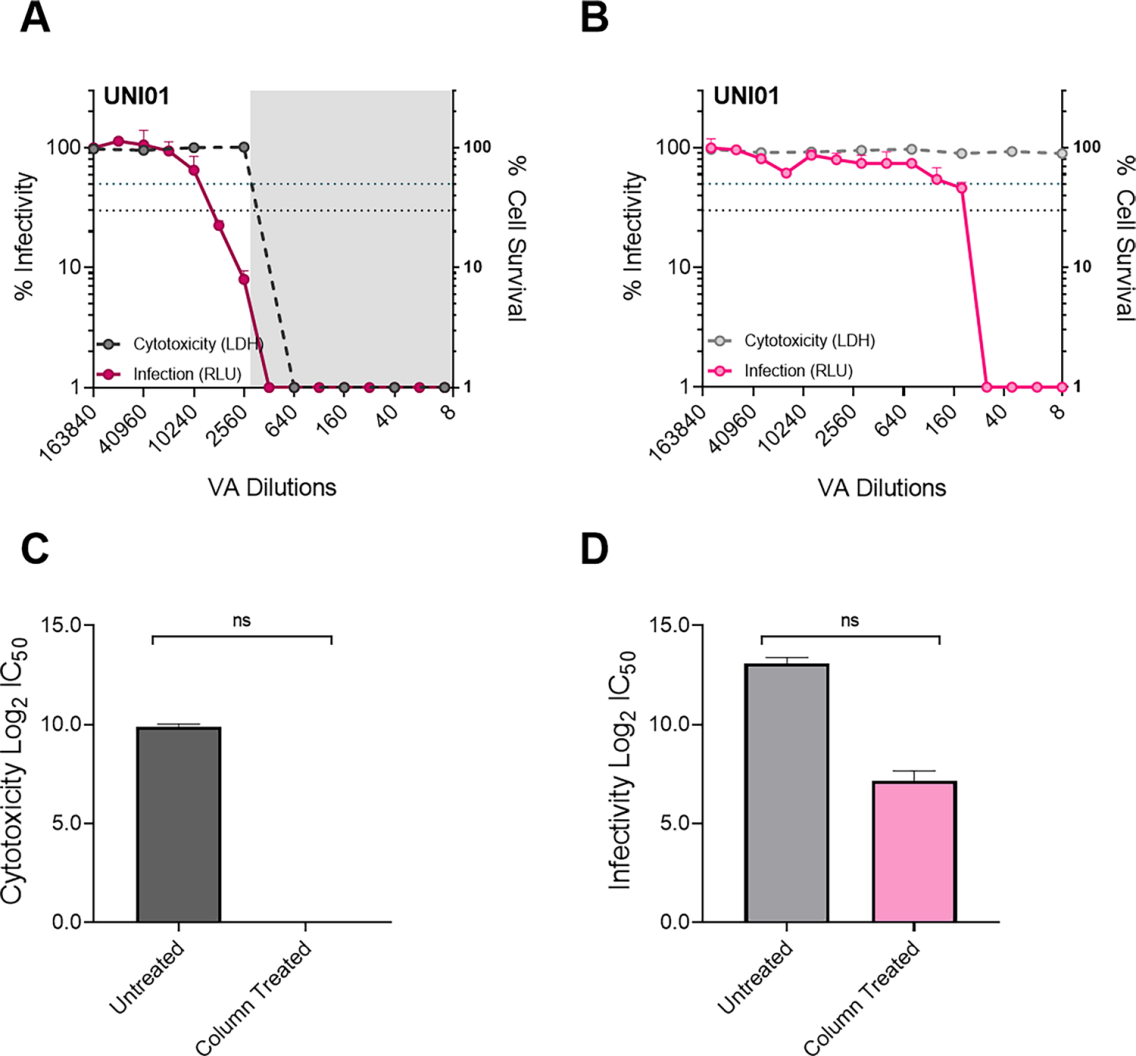
Comparison of UNI01 virucidal activity assay with and without deactivation of cytotoxicity using Cytiva Microspin S-400 HR columns. In all cases, SARS-CoV-2 S enveloped PV was used to transduce 293T ACE2 TMPRSS2 to determine infectivity, expressed as relative light units (RLU), and LDH assay was used to determine cytotoxicity of the same cells. **(A)** Infectivity and cytotoxicity curves for UNI01 virucidal activity assay without removal of cytotoxicity (n = 2). **(B)** Infectivity and cytotoxicity curves for UNI01 virucidal activity assay with removal of cytotoxicity (n = 2). **(C)** Comparison of Log2 IC_50_ values for UNI01 cytotoxicity with and without removal of cytotoxicity (n = 2). **(D)** Comparison of Log2 IC_50_ values for UNI01 virucidal activity with and without removal of cytotoxicity (n = 2). For cytotoxicity graphs, the shaded area represents zone in which reagent demonstrates over 50% cytotoxicity whilst the blue dotted line represents 50% cytotoxicity and virucidal activity and black dotted line represents 70% cytotoxicity and virucidal activity. Significance determined by Wilcoxon test.

We next wanted to determine the effect of high cytotoxicity and removal of cytotoxicity on the outcome of the virucidal activity assay. To achieve this, we compared the reduction in infectivity when the PV/VA mix was prepared prior to treatment with the Cytiva Microspin S-400 HR column to a condition without column treatment. We initially compared virucidal activity measurements in treated and untreated preparations of UNI01 and SARS-CoV-2 (S) enveloped PV. We showed that the virucidal activity curve was shifted to a higher dilution and that there was substantial overlap with cell survival when the cytotoxicity was not removed from the preparation (Fig. 4A), as compared to the condition in which cytotoxicity was removed (Fig. 4B). The overlap between cell survival and virucidal activity indicates that cytotoxicity has a considerable impact on the measurement of virucidal activity. There was a substantial, though not significant, difference between treated and untreated conditions when measuring the 50% cytotoxicity (Fig. 4C) and 50% virucidal activity (Fig. 4D). Specifically, without removing cytotoxicity, the Log2 IC_50_ for UNI01 for deactivation of SARS-CoV-2 (S) enveloped PV was 13.1 (corresponding to a 1/8777 dilution) compared to 7.16 (corresponding to a 1/144 dilution) when cytotoxicity is removed (Fig. 4D).

**Figure 5:**
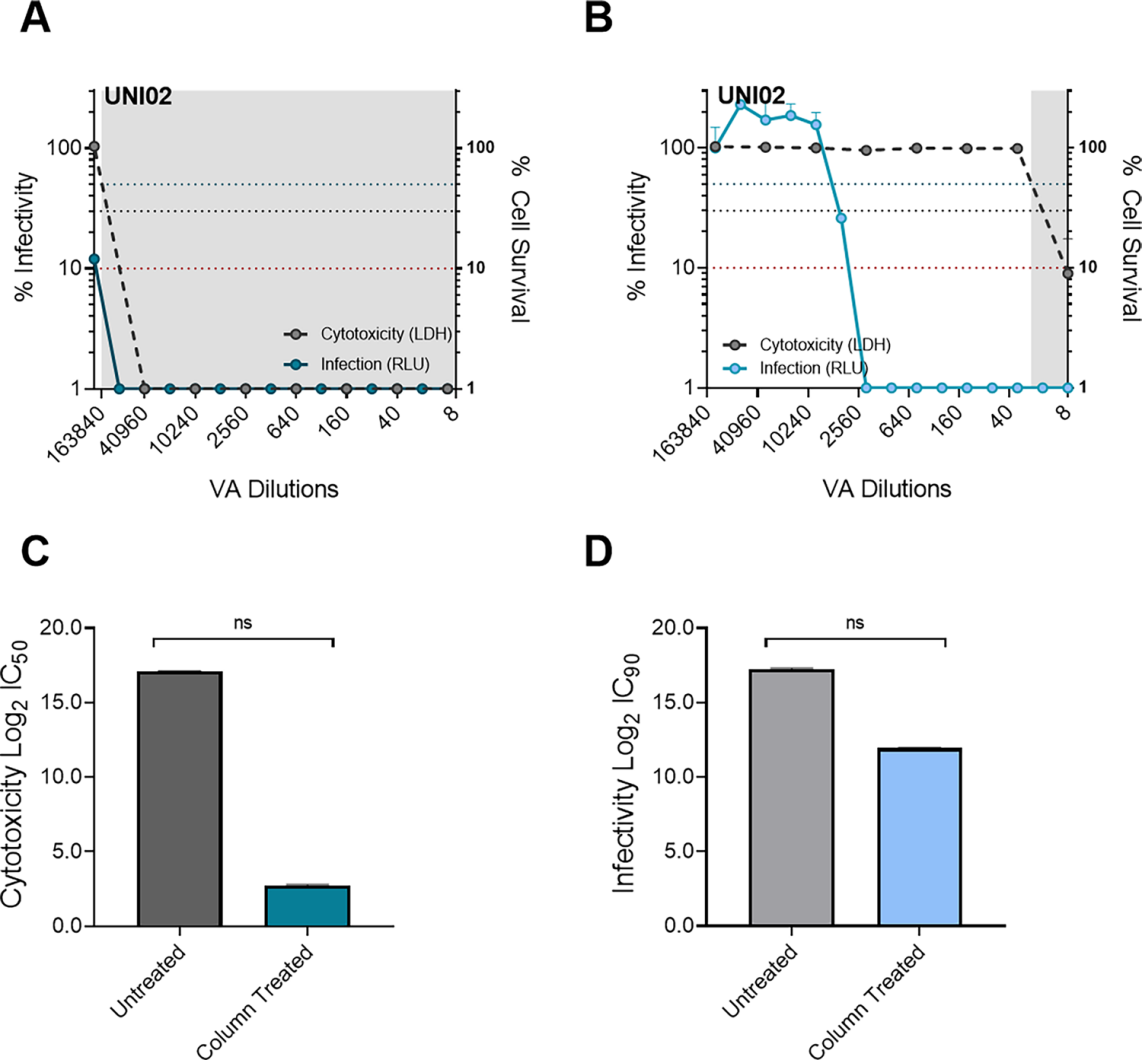
Comparison of UNI02 virucidal activity assay with and without deactivation of cytotoxicity using Cytiva Microspin S-400 HR columns. In all cases, SARS-CoV-2 S enveloped PV was used to transduce 293T ACE2 TMPRSS2 to determine infectivity, expressed as relative light units (RLU), and LDH assay was used to determine cytotoxicity of the same cells. **(A)** Infectivity and cytotoxicity curves for UNI02 virucidal activity assay without removal of cytotoxicity (n = 2). **(B)** Infectivity and cytotoxicity curves for UNI02 virucidal activity assay with removal of cytotoxicity (n = 2). **(C)** Comparison of Log2 IC_50_ values for UNI02 cytotoxicity with and without removal of cytotoxicity (n = 2). **(D)** Comparison of Log2 IC_90_ values for UNI02 virucidal activity with and without removal of cytotoxicity (n = 2). For cytotoxicity graphs, the shaded area represents zone in which reagent demonstrates over 50% cytotoxicity whilst the blue dotted line represents 50% cytotoxicity and virucidal activity, black dotted line represents 70% cytotoxicity and virucidal activity and the red dotted line represents 90% virucidal activity. Significance determined by Wilcoxon test.

Similarly, we performed this comparison for the virucidal activity of UNI02 against SARS-CoV-2 (S) enveloped PV. Again, we showed that the virucidal activity curve was shifted to a far higher dilution and that there was substantial overlap with cell survival when the cytotoxicity was not removed from the preparation (Fig. 5A), as compared to the condition in which cytotoxicity was removed (Fig. 5B). As with the previous result, the overlap between cell survival and virucidal activity indicates that cytotoxicity has a substantial impact on the measurement of virucidal activity. There was a substantial, though not significant, difference between treated and untreated conditions when measuring the 50% cytotoxicity (Fig. 5C) and 50% virucidal activity (Fig. 5D). Specifically, we showed that without removing cytotoxicity, the Log2 IC_90_ for UNI02 was 17.23 (corresponding to 1/115,386 dilution) compared to 11.94 (corresponding to 1/3948 dilution) when cytotoxicity is removed (Fig. 5D).

### Deactivation of highly cytotoxic reagents

**Figure 6:**
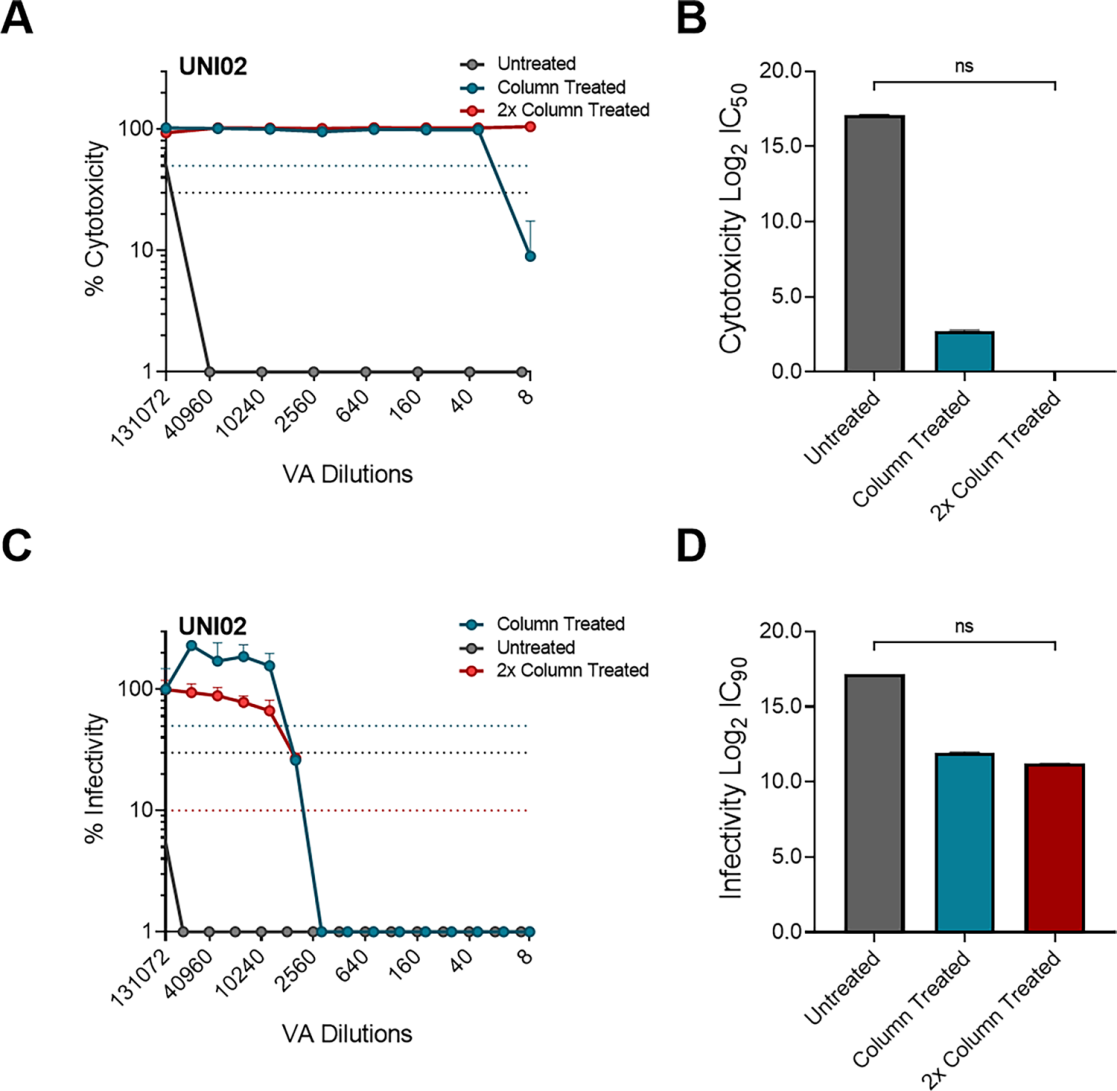
Enhanced removal of cytotoxicity using 2 × Cytiva Microspin S-400 HR column treatment. **(A)** Cytotoxicity curve comparing UNI02 cytotoxicity when there is either; no treatment (grey), treatment by passing through column once (blue) or passing through column twice (red) (n = 2). **(B)** Corresponding IC_50_ measurements of cytotoxicity when with three different treatments (n = 2). **(C)** Virucidal activity curve comparing EB virucidal activity when there is no treatment (grey), treatment by passing through column once (blue) or passing through column twice (red) (n = 2). **(D)** Corresponding IC_50_ measurements of virucidal activity when with the three different treatments (n = 2). For cytotoxicity and virucidal activity, the blue dotted line represents 50% inhibitory concentration, the black dotted line represents 70% inhibitory concentration and the red dotted line represents 90% inhibitory concentration. Significant difference between IC_50_ values determined by Kruskal–Wallis test with Dunn’s multiple comparisons.

Due to the examples of incomplete removal of cytotoxicity for UNI02 (Fig. 2B) and UNI04 (Fig. 2D), we aimed to develop a method that could be used to remove 100% of cytotoxicity in these products whilst still retaining the sensitivity required to measure virucidal activity. To this end, we measured the removal of cytotoxicity of UNI02 when the PV/VA mix was passed through a Cytiva Microspin S-400 HR column and then the 100 µl flow through was re-applied to another column, after which the flow-through was diluted 1/5 in complete DMEM. Through this method, we demonstrated complete removal of cytotoxicity of UNI02, with 100% cell survival (0% cytotoxicity) at the highest VA concentration (Fig. 6A, B). Next, to determine if the PV could be recovered and remained infectious following this process, we once again measured the PV concentration and infectiousness after being passed through the columns twice. Further, in order to determine the viability of this method for lower titre PV stocks or when using envelopes with inherently lower infectivity, this analysis was performed with two different PV input dilutions: undiluted PV and PV that was diluted 1/5. For undiluted PV input, PV was still detectable via p24 capsid ELISA, with 84 ng/ml p24 in the input PV resulting in recovery of 42 ng/ml p24 when passed through the column once and 29 ng/ml when passed through the column twice (Suppl. Fig. 3A). Similarly, the infectivity of the PV flow-through was still detectable when passed through the column twice, going from 5.8 × 10^5^ RLU in input PV, to 2.4 × 10^5^ and 1.1 × 10^5^ when the PV was passed through the column once and twice, respectively (Suppl. Fig. 3B). These results demonstrate that high titre (undiluted) PV can be used to measure virucidal activity using this method. For diluted PV, representing low titre or low infectivity PV, we showed that PV was still detectable following treatment through the column twice, reducing from 46 ng/ml for input PV to 9.5 ng/ml and 5.9 ng/ml when the PV was passed through the column once and twice, respectively (Suppl. Fig. 3C). Similarly, PV infectivity was low, but still detectable, after being passed through a column twice, going from 3.0 × 10^5^ RLU for input PV to 5.4 × 10^5^ and 2.3 × 10^5^ RLU when passed through the column once and twice, respectively (Suppl. Fig. 3D).

Finally, we aimed to test the enhanced cytotoxicity removal method for its ability to determine the virucidal activity of UNI02 against SARS-CoV-2 (S) enveloped PV. We showed similar virucidal activity curves for both the 1× column treated and 2× column treated conditions (Fig. 6C). Indeed, the Log2 IC_90_ of UNI02 was close to identical when treated with the column 1× (11.94.33, corresponding to a dilution of 1/3948) and 2× (11.22, corresponding to a dilution of 1/2385) (Fig. 6B). Together, these results indicate that, in the case of highly cytotoxic products, passing through a column twice should remove 100% cytotoxicity. In most cases, this method retains enough sensitivity to measure virucidal activity, however, for lower input values this method may not be appropriate and further investigation is required to determine the lower limit of detection.

## Discussion

The outbreak of SARS-CoV-2 in late 2019 and its rapid global expansion has highlighted significant deficiencies in the global response to viral disease outbreaks. This, coupled with the recent increase in the outbreak of viral zoonoses^1–3^, indicates that viral pandemics are a major threat to global health. Inactivation of viruses using virucidal reagents is the first line of defence deployed to prevent the spread of viral diseases, particularly during the early stages of outbreaks and in the absence of pharmaceutical interventions. In this study, we have used pseudo-typed virus particles to develop a fast and straightforward method to evaluate the efficacy of different virucidal reagents and precisely quantify their minimum inhibitory concentrations.

Different viruses exhibit variation in their resistance to inactivation using chemical germicides, with non-enveloped viruses generally being the most resistant to inactivation due to the requirement of the virucidal agent to denature the protein capsid^51^. Conversely, enveloped viruses are more susceptible to inactivation and only require disruption of the lipid envelope to prevent virus infectivity^23,51,52^. In the case of enveloped viruses, current European guidelines (EN 14476) to assess the efficacy of virucidal reagents against viruses in suspension endorse the use of MVA as a safe surrogate for the virus of interest^52,53^, on the basis that MVA is one of the most inactivation resistant enveloped viruses^30,54^. Otherwise, the virus of interest itself can be used in live virus assays to measure the precise susceptibility of that virus to chemical inactivation, as has been used previously for SARS-CoV-2^24–26,28^. In this present study, we have used PV as a surrogate for live viruses based on the rationale that PVs exhibit the basic structure of enveloped viruses and enable the expression of a range of viral envelope proteins. Therefore, it is likely that the inactivation susceptibility of PVs is comparable to that of other enveloped viruses. Indeed, a previous study has demonstrated comparable levels of susceptibility to ozone mediated inactivation between coronavirus enveloped PV and live Human Coronavirus-229E (HuCoV-229E)^55^. Nevertheless, the PV that was used in this proposed assay comprises the core of HIV-1 which is among the least inactivation resistant enveloped viruses^23^. As such, the use of PV as a surrogate for live virus may not support the claim that the virucidal agent deactivates all enveloped viruses.

Despite this, the major benefit of this PV based assay is the relative low cost, the short turnaround time and simple quantitative output. As previously discussed, live-virus assays are commonly used to determine virus inactivation, though this presents technical limitations due to the requirement for established virus culture methods and high containment facilities. While the use of MVA to assess virucidal activity overcomes the issues associated with using the specific virus of interest, these assays require long culture times^30,54^ and are therefore less suited to studies that aim to optimise disinfectant formulations by determining the inhibitory concentrations of individual virucidal components. Here, we have developed a method that can determine the inhibitory concentrations (IC_90_) of different virucidal reagents in 48 h and using an assay with a simple quantitative output, with the view that this assay can be used as a preliminary step to optimise disinfectant formulations and assess synergy between different formula components.

An important consideration for the development of cell-based assays to measure the efficacy of virucidal agents is the cytotoxicity of the formulation. We showed that UNI01, UNI02 and UNI04 were highly cytotoxic (Fig. 1) and hypothesised that this high level of cytotoxicity is likely to impair the ability to accurately quantify virus inhibition. Other assays that use live virus to measure virus inactivation often rely on substantial dilution of the virus and virucidal agent mix or dilution of this mix in a neutralising broth to effectively eliminate the associated cytotoxicity^23–26,28–30,48^. However, this approach is less feasible when using non-replicative PVs, as they do not undergo multiple rounds of infection and therefore follow linear infection kinetics, limiting the overall sensitivity of detection. As such, we developed an approach to remove cytotoxicity using a combination of treatment with Cytiva Microspin S-400 HR columns and a small dilution in complete DMEM. We showed that this approach removed the majority of cytotoxicity from the four VAs tested (Fig. 2) while retaining enough sensitivity to assay the reduction in infectivity (Supplementary Fig. 1). Additionally, we demonstrated that the removal of cytotoxicity was necessary to accurately determine the minimum inhibitory concentrations of highly cytotoxic reagents, resulting in substantial, though non-significant, differences in the measured inhibitory concentrations when comparing the assay with or without the removal of cytotoxicity (Figs. 4 and 5). It is likely that the lack of significant differences observed was due to the low number of replicates used to determine inhibitory concentrations. Nevertheless, the removal of cytotoxicity is an essential step for studies that aim to optimise the formulation of different disinfectants and individual VAs. We have also provided a method to enhance the removal of cytotoxicity in cases where the VA was highly cytotoxic, although we showed that this was not necessary to accurately determine the virucidal concentrations of this reagent (Fig. 6). Despite this, the method was only tested on one product, and further investigation is required to determine if it is applicable to other highly cytotoxic compounds. As well as this, the lowest PV input tested in this study was 46 ng/ml p24, and therefore the lowest limit of detection for this method is unknown and it may not be sensitive to allow detection of very low titre PV.

We compared the susceptibility to inactivation of PVs produced with envelopes from different viruses and showed that there was no significant difference between HIV-1 (X4), SARS-CoV-2 (S) and Influenza A/Indonesia (H5) when they were treated with UNI01 and UNI02 (Fig. 3). This is likely because the majority of virucidal activity is provided by the disruption of the virus lipid envelope, which is the same among all the PVs in this study, rather than by denaturing or deactivating specific envelope proteins. Nevertheless, the method for production of PVs is similar or identical when producing PVs with envelope proteins from different viruses, therefore, this method can be easily adapted to measure virucidal activity against a range of viruses.

In this study, we have provided a method to measure the virus inhibition range of different virucidal agents against enveloped viruses in suspension. Further, this method could be readily adapted to include interfering substances to comply with phase 2, step 1 of the European guidelines to measure virucidal activity^23,52^. Whilst this method may not provide the ability to indicate inactivation of all enveloped viruses, the relative speed and simple quantitative output of this method means that it is a useful tool to optimise the use of VAs in different disinfectant formulations.

### Supplementary Information

Supplementary Figures.

## Data Availability

The raw data supporting the conclusions of this article will be made available by the authors, without undue reservation by contacting J.T or W.A.P.
