# Supplementary material for: Development of a pseudo-typed virus particle based method to determine the efficacy of virucidal agents
Source: Sci Rep. 2024 Jan 25;14:2174. doi: 10.1038/s41598-024-52177-2 (PMC10810821; doi:10.1038/s41598-024-52177-2)
Supplement: Supplementary file 1 — Supplementary Figures. [file 41598_2024_52177_MOESM1_ESM.pdf]

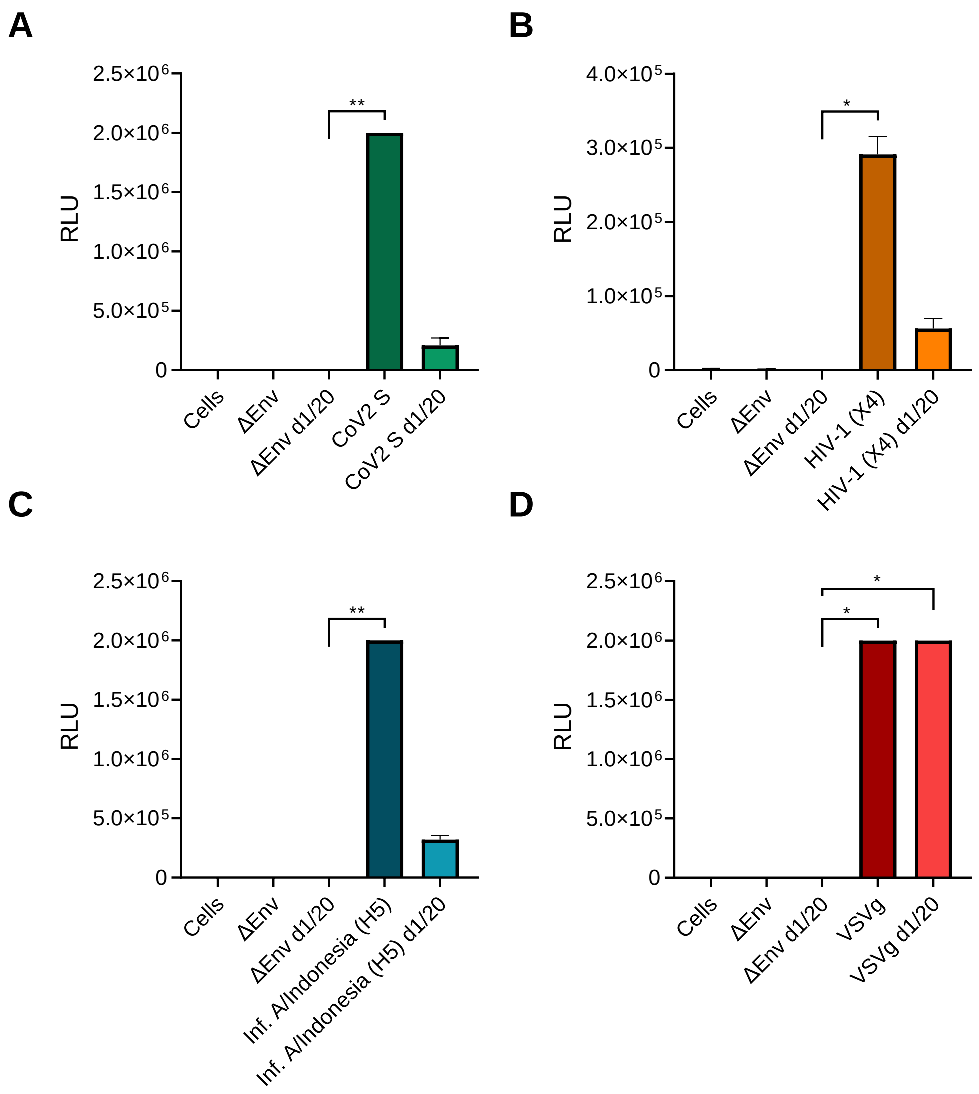

Supplementary Figure 1: Production of variant enveloped PV stocks for use in virus deactivation assays. In all cases, PV infection was measured using transduction of virus specific cell types with a cell only negative control as well as an enveloped negative (ΔEnv) control that was produced using the same method and during the same transfection of the measured PV. Both PV and ΔEnv were measured neat (left and darker bar) and 1 in 20 diluted (right and lighter bar, with d1/20 label). **A)** Transduction of 293T ACE2 TMPRSS2 cells with SARS-CoV-2 and non-enveloped PV (n=6). **B)** Transduction of TZMbl cells with HIV-1 LAI (X4) and non-enveloped PV. **C)** Transduction of 293T cells with Influenza A/Indonesia and non-enveloped PV. **D)** Transduction of 293T ACE2 TMPRSS2 cells with VSVg and non-enveloped PV. Significance determined by Kruskal-Wallis test with Dunn’s multiple comparisons.

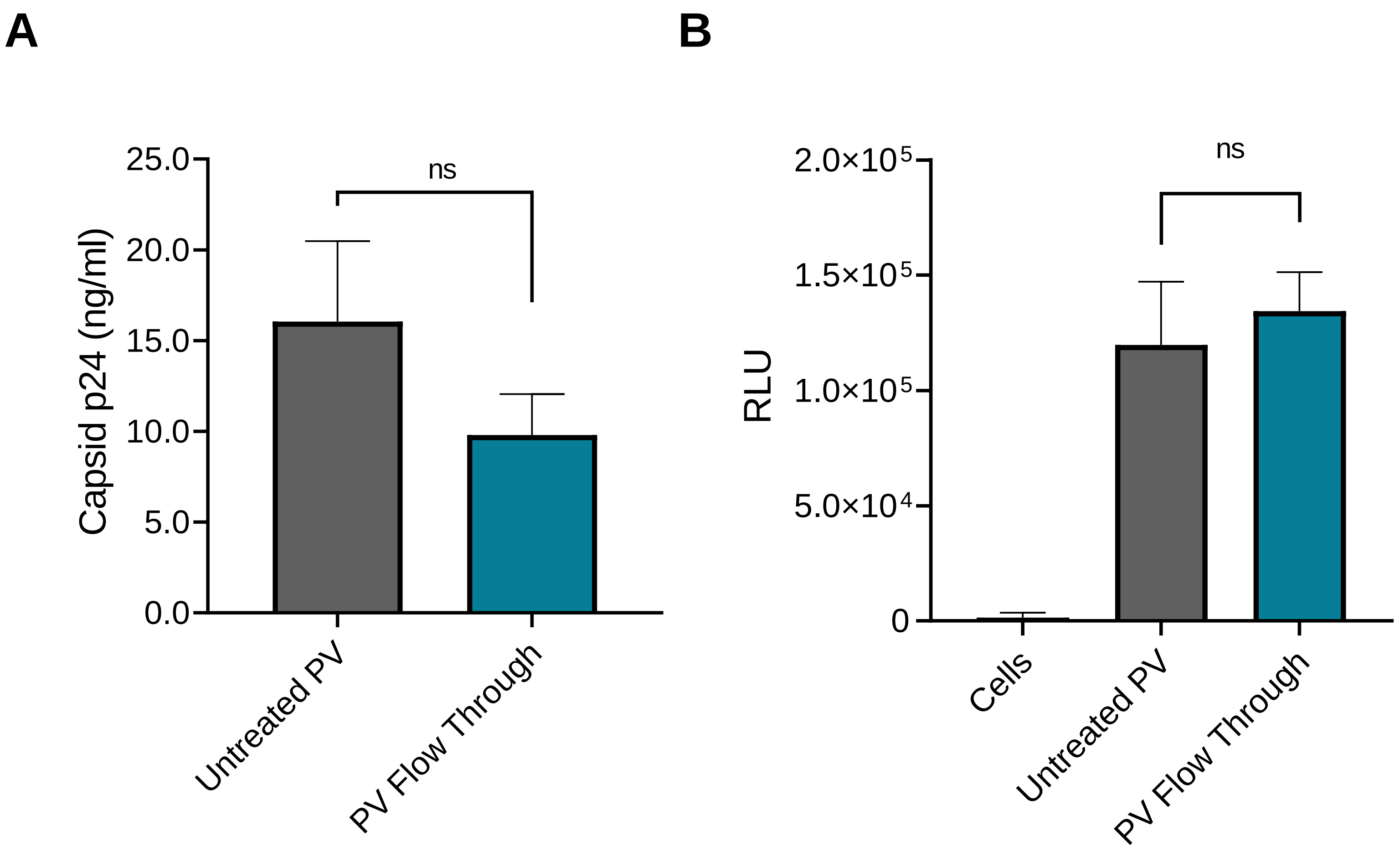

Supplementary Figure 2: PV recovery from Cytivia Microspin S-400 HR column treatment as measured by quantitation of PV through ELISA and determination of infectiousness. A) Measurement of HIV-1 capsid p24 from input PV (grey) and from PV that has been passed through a Cytivia Microspin S-400 HR column (blue). **B)** Measurement of PV infectivity from uninfected cells, input PV (grey) and PV that has been passed through a Cytivia Microspin S-400 HR column (blue), with infectivity expressed as relative light units (RLU). Significance determined by Wilcoxon test.

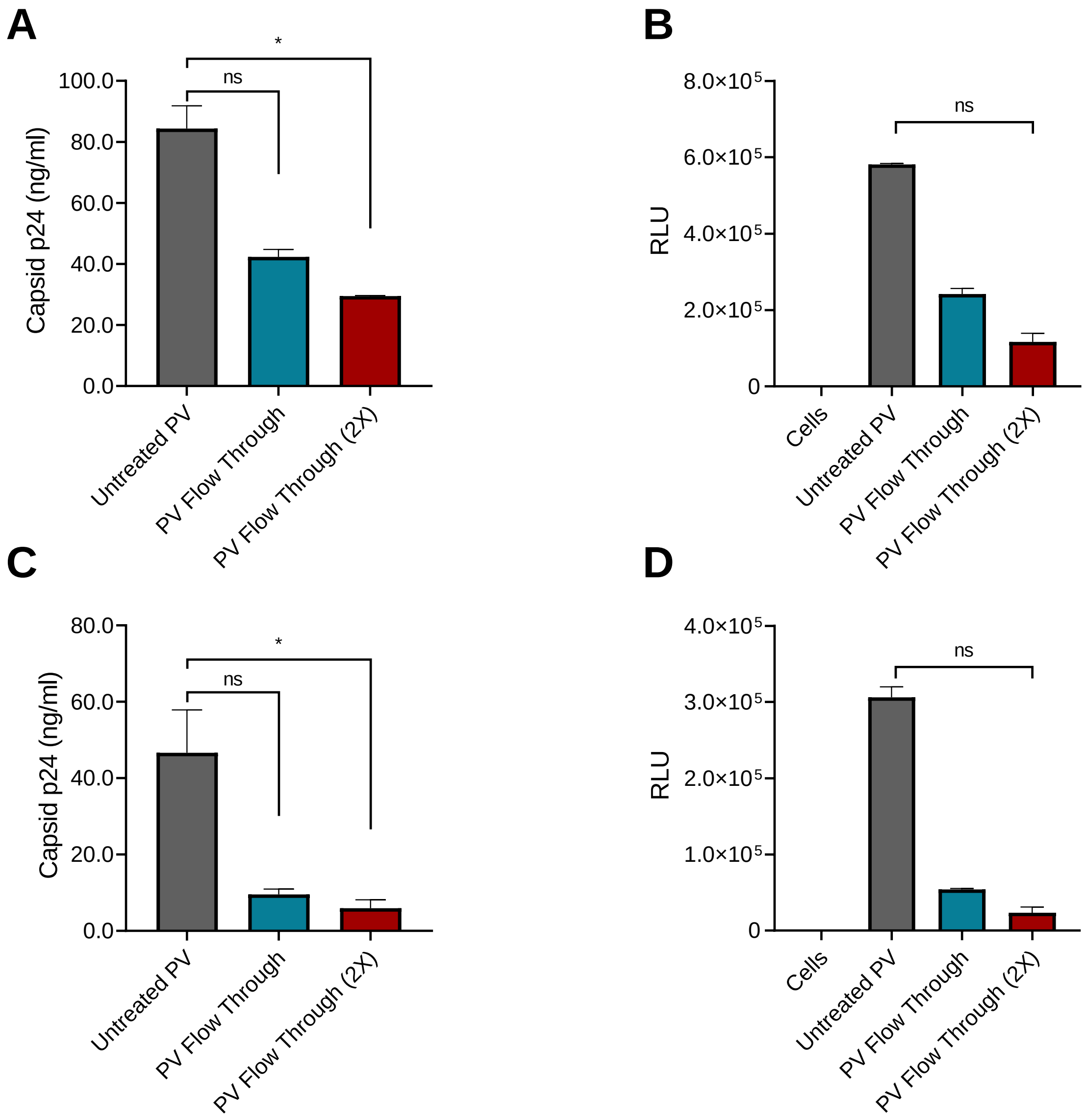

Supplementary Figure 3: PV recovery from 1x and 2x Cytivia Microspin S-400 HR column treatment as measured by quantitation of PV through ELISA and determination of infectiousness. A) Comparison of recovery of undiluted PV without treatment (grey), when passed through a column once (blue) and twice (red), as measured by capsid p24 ELISA. **B)** Recovery of infectivity of undiluted PV without treatment (grey), when passed through a column once (blue) and twice (red), as measured by luciferase activity and expressed as RLU. **C)** Comparison of recovery of PV diluted 1/5 without treatment (grey), when passed through a column once (blue) and twice (red), as measured by capsid p24 ELISA. **D)** Recovery of infectivity of PV diluted 1/5 without treatment (grey), when passed through a column once (blue) and twice (red), as measured by luciferase activity and expressed as relative light units (RLU). Significant difference between IC_{50} values determined by Kruskal-Wallis test with Dunn's multiple comparisons.
